# Supplementary material for: Development of A4 antibody for detection of neuraminidase I223R/H275Y-associated antiviral multidrug-resistant influenza virus
Source: Nat Commun. 2020 Jul 9;11:3418. doi: 10.1038/s41467-020-17246-w (PMC7347576; doi:10.1038/s41467-020-17246-w)
Supplement: Supplementary file 1 — Supplementary Information [file 41467_2020_17246_MOESM1_ESM.pdf]

Supplementary Information

Development of A4 Antibody for Detection of Neuraminidase I223R/H275Y-Associated Antiviral Multidrug-Resistant Influenza Virus

Kyeonghye Guk[1,2], Hyeran Kim[1], Miyeon Lee[3], Yoon-Aa Choi[4], Seul Gee Hwang[1,2], Gaon Han[1,2], Hye-Nan Kim[1], Hongki Kim[1], Hwangseo Park[5], Dongeun Yong[6], Taejoon Kang[1*], Eun-Kyung Lim[1,2*], and Juyeon Jung[1,2*]

[1]Bionanotechnology Research Center, Korea Research Institute of Bioscience & Biotechnology (KRIBB), 125 Gwahak-ro, Yuseong-gu, Daejeon 34141, Republic of Korea

[2]Deparment of of Nanobiotechnology, KRIBB School of Biotechnology, University of Science and Technology (UST), 217 Gajeong-ro, Yuseong-gu, Daejeon 34113, Republic of Korea

[3]Department of Chemistry, Korea Advanced Institute of Science and Technology (KAIST), 291 Daehak-ro, Yuseong-gu, Daejeon 34141, Republic of Korea

[4]BioNano Health Guard Research Center, KRIBB, 125 Gwahak-ro, Yuseong-gu, Daejeon 34141, Republic of Korea

[5]Department of Bioscience and Biotechnology, Sejong University, 209 Neungdong-ro, Kwangjin-gu, Seoul 05006, Republic of Korea

[6]Department of Laboratory Medicine and Research Institute of Bacterial Resistance, Yonsei University College of Medicine, Seoul 03722, Republic of Korea

[*]E-mail: jjung@kribb.re.kr (J.J.); eklim1112@kribb.re.kr (E-K.L.); kangtaejoon@kribb.re.kr (T.K.)

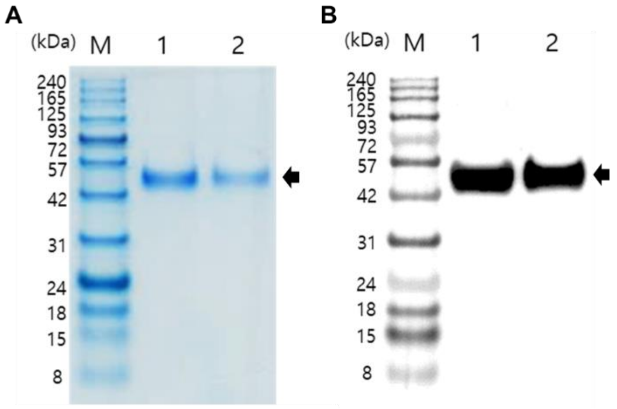

Supplementary Figure 1. Identification of wt NA and I223R/H275Y NA by (A) SDS-PAGE and (B) Western blot analysis. 3 times each experiment was repeated independently with similar results. Lane M: protein marker; Lane 1: wt NA; Lane 2: I223R/H275Y NA.

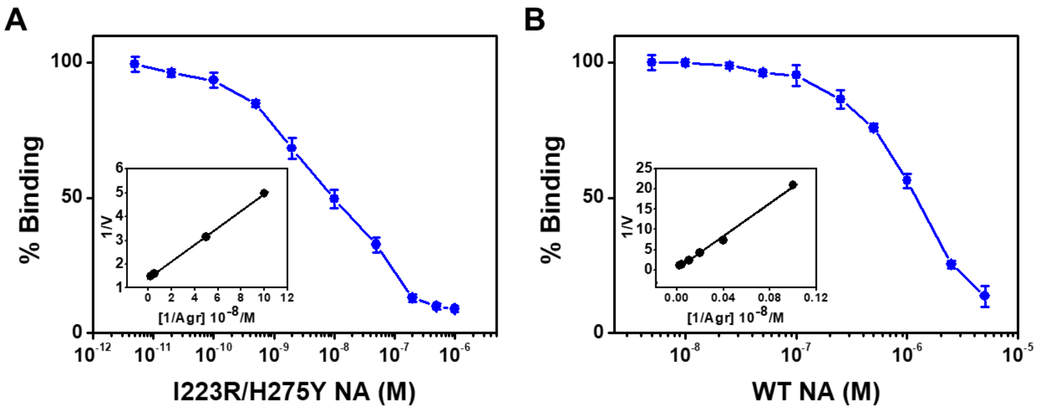

Supplementary Figure 2. (A) Binding activity of A4 to I223R/H275Y NA by competition ELISA. (B) Binding activity of A4 to wt NA by competition ELISA. Error bars = standard deviation (n=3).

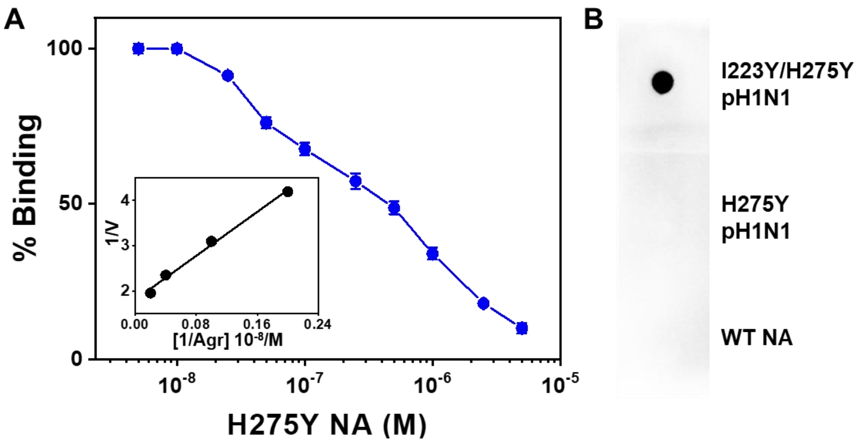

Supplementary Figure 3. (A) Binding activity of A4 to H275Y NA by competition ELISA. Error bars = standard deviation (n=3). (B) Interaction of A4 to I223Y/H275Y pH1N1 (10^7 PFU mL^{-1}), H275Y pH1N1 (10^7 PFU mL^{-1}), and wt NA (0.5 mg mL^{-1}) by dot-blot analysis. 3 times each experiment was repeated independently with similar results.

```
Light chain
G6          GAMAQPAHGSGDIQMTQSPSSLSASVGDRVTITCRASQDVSTAVAWYQQKPGKAPKLLIY   60
A4          -----------DIQMTQSPSSLSASVGDRVTITCRASQSVSNWLAWYQQKPGKAPKLLIY   49

G6          SASFLYSGVPSRFSGSGSGTDFTLTISSLQPEDFATYYCQQHYTTPPTFGCGTKVEIKGG   120
A4          WASSLESGVPSRFSGSGSGTDFTLTISSLQPEDFATYYCQQYYNHPYTFGQGTKVEIK--   107

Heavy chain
G6          EVQLVESGGGLVQPGGSLRLSCAASGFTISDYWIHWVRQAPGKCLEWVAGITPAGGYTYY   60
A4          EVQLVESGGGLVQPGGSLRLSCAASGFTFNSYWMSWVRQAPGKGLEWVSWITPTSGTTYY   60

G6          ADSVKGRFTISADTSKNTAYLQMNSLRAEDTAVYYCARFVFF--LPYAMDYWGQGTLVTV   118
A4          ADSVKGRFTISRDNSKNTLYLQMNSLRAEDTAVYYCARVEDWDIGYYGMDYWGQGTLVTV   120

G6          SSASAAAGGGLNDIFEAQKIEWHE   142
A4          SSAS--------------------   124
```

Supplementary Figure 4. Alignment of amino acid sequences of A4 with respect to homologous antibody G6 for which X-ray crystal structure is known. The identical amino acids among the three antibodies are indicated in red.

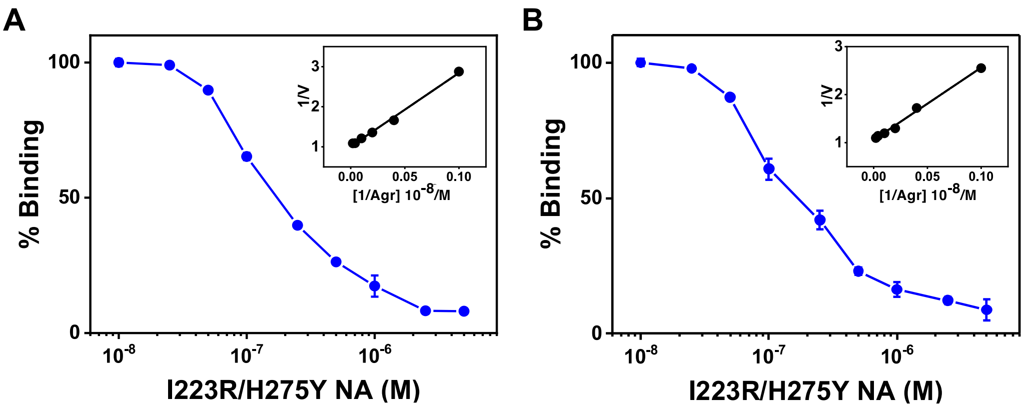

Supplementary Figure 5. (A) Binding activity of H94A mutant A4 to I223R/H275Y NA by competition ELISA. (B) Binding activity of W33A mutant A4 to I223R/H275Y NA by competition ELISA. Error bars = standard deviation (n=3).

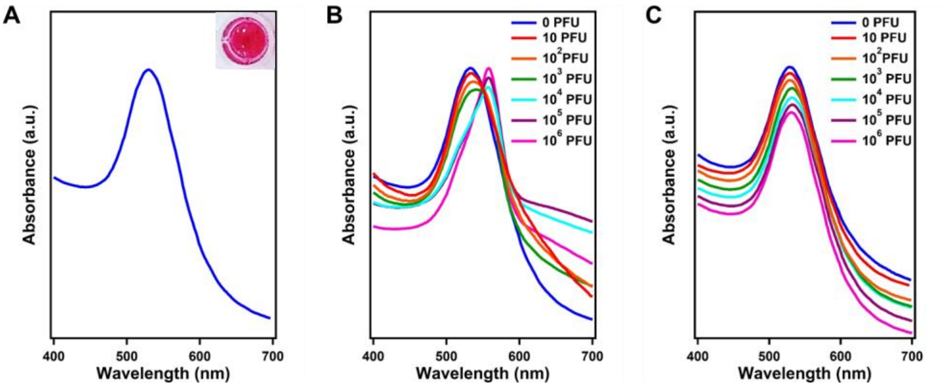

Supplementary Figure 6. (A) Absorption spectrum of A4-Au NPs. Inset is optical image of A4-Au NPs. (B) Absorption spectra of A4-Au NPs in the presence of I223R/H275Y pH1N1. (C) Absorption spectra of A4-Au NPs in the presence of wt pH1N1. 3 times each experiment was repeated independently with similar results.

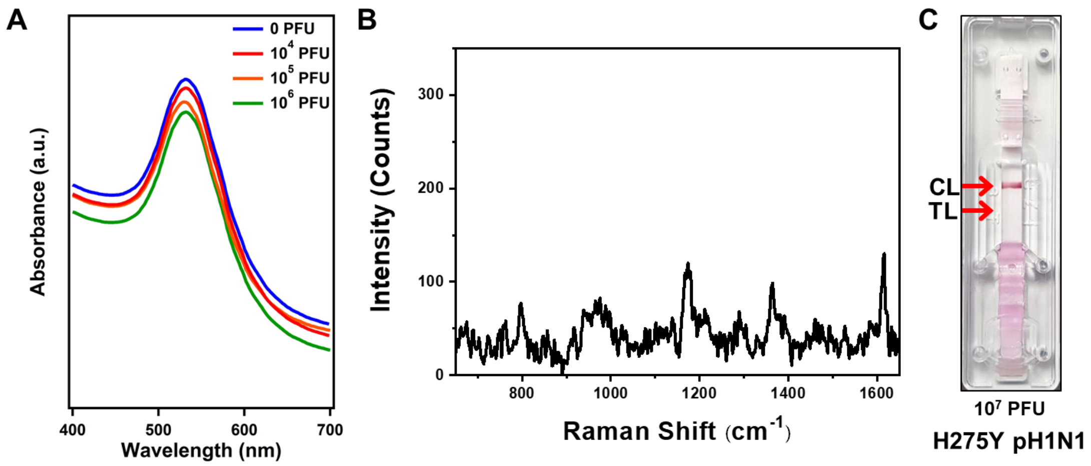

Supplementary Figure 7. (A) Absorption spectra of A4-Au NPs in the presence of H275Y pH1N1. 3 times each experiment was repeated independently with similar results. (B) SERS spectra of MGITC obtained from NPs-on-plate structures in the presence of H275Y pH1N1 (10^6 PFU). 10 times each experiment was repeated independently with similar results. (C) Optical image of A4-based lateral flow system after detection of H275Y pH1N1 (10^7 PFU).

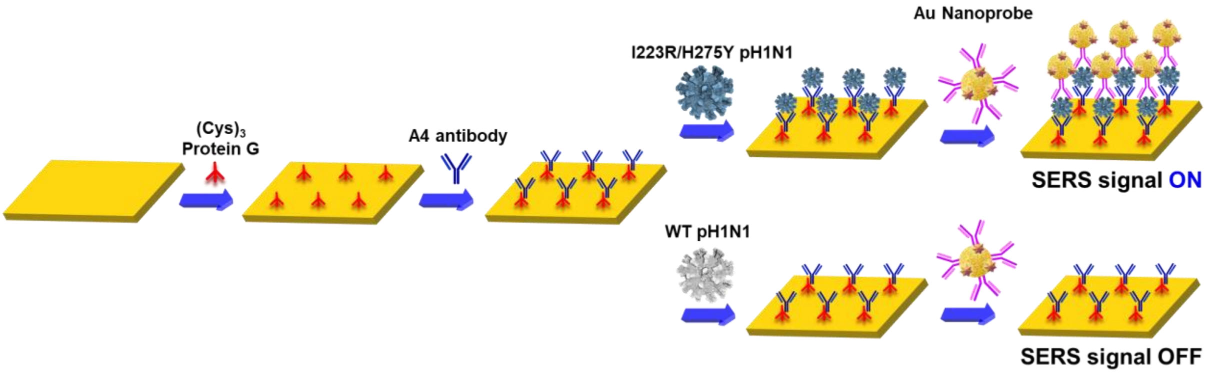

Supplementary Figure 8. Schematic illustration of SERS-based immunoassay for I223R/H275Y pH1N1 using A4.

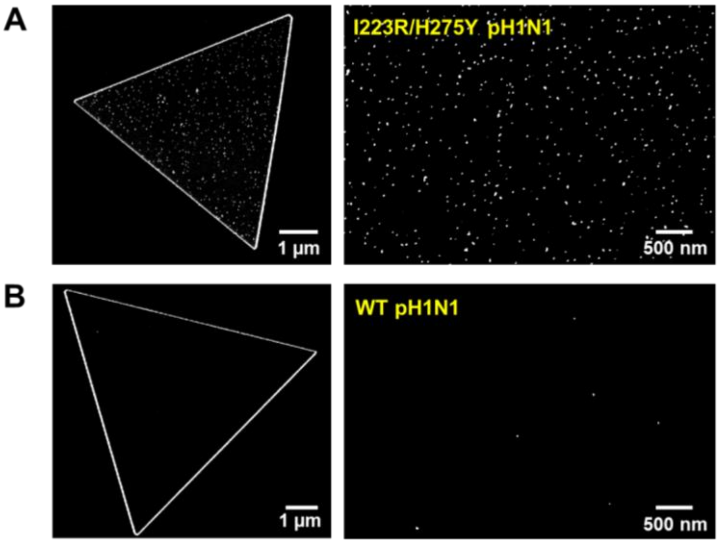

Supplementary Figure 9. SEM images of NPs-on-plate structures after incubating in (A) I223R/H275Y pH1N1 and (B) wt pH1N1. The number of both viruses is 1,500 PFU. 10 times each experiment was repeated independently with similar results.

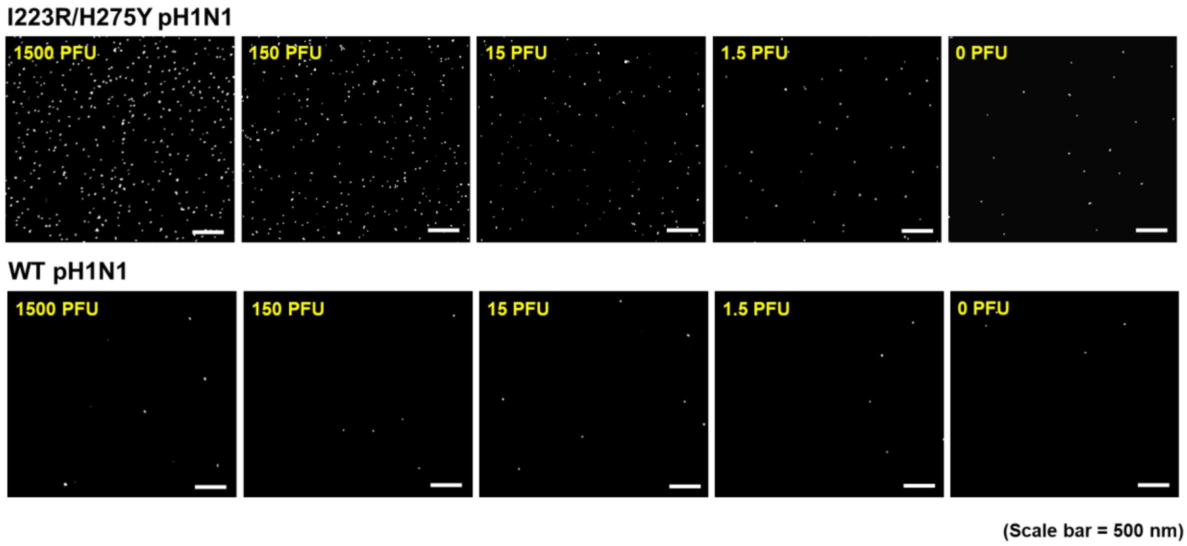

Supplementary Figure 10. SEM images of NPs-on-plate structures after incubating in I223R/H275Y pH1N1 (upper panel) and wt pH1N1 (lower panel). The number of both viruses is varying from 0 to 1,500 PFU. 10 times each experiment was repeated independently with similar results.

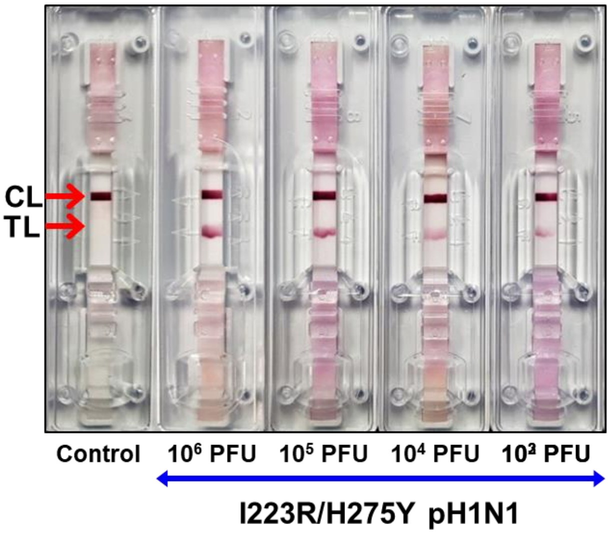

Supplementary Figure 11. Optical images of A4-based lateral flow systems after detection of I223R/H275Y pH1N1in nasopharyngeal swab and control samples.

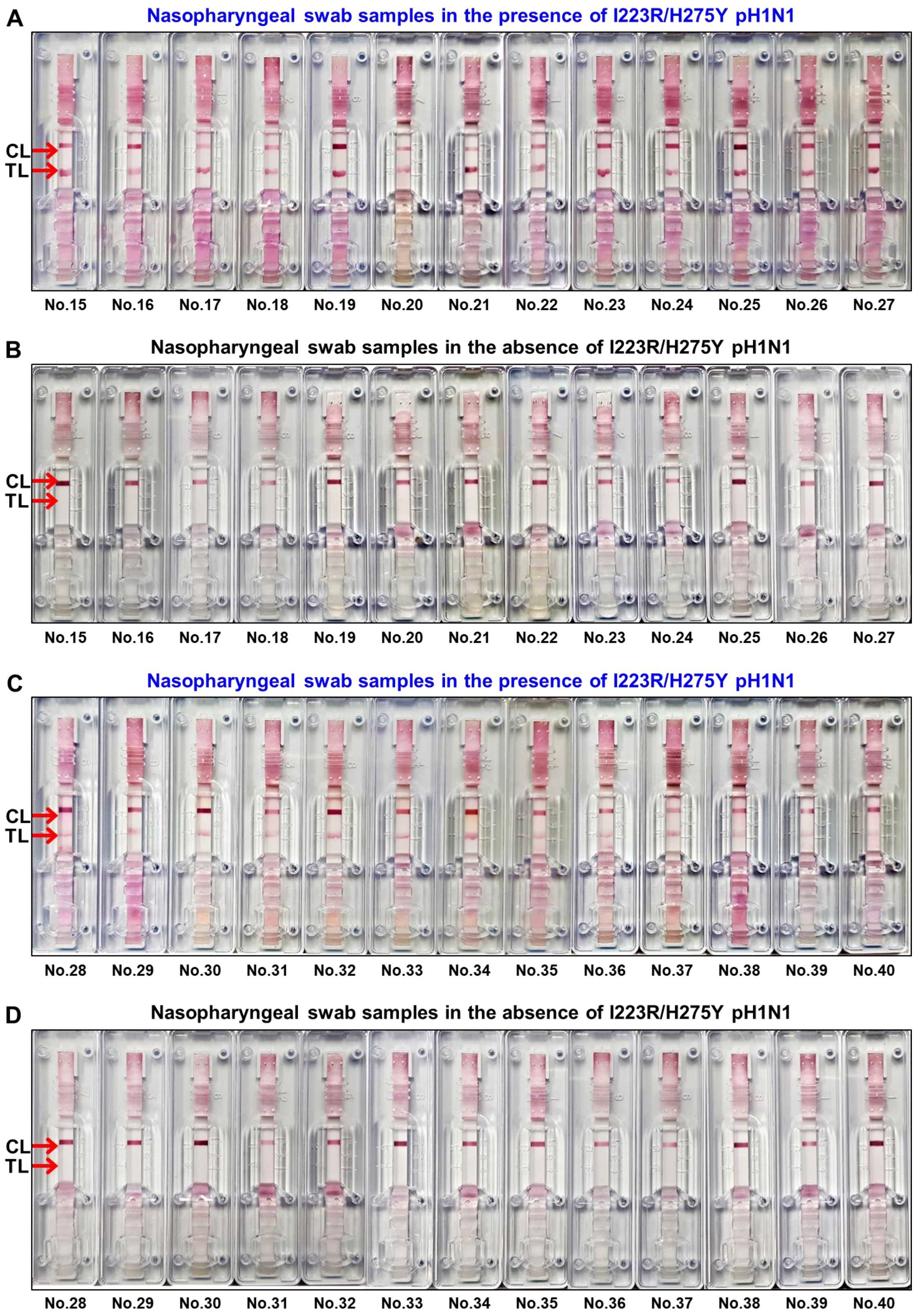

Supplementary Figure 12. Optical images of A4-based lateral flow systems after detection of nasopharyngeal swab samples (A,C) in the presence or (B,D) absence of I223R/H275Y pH1N1 virus (10^3 PFU).

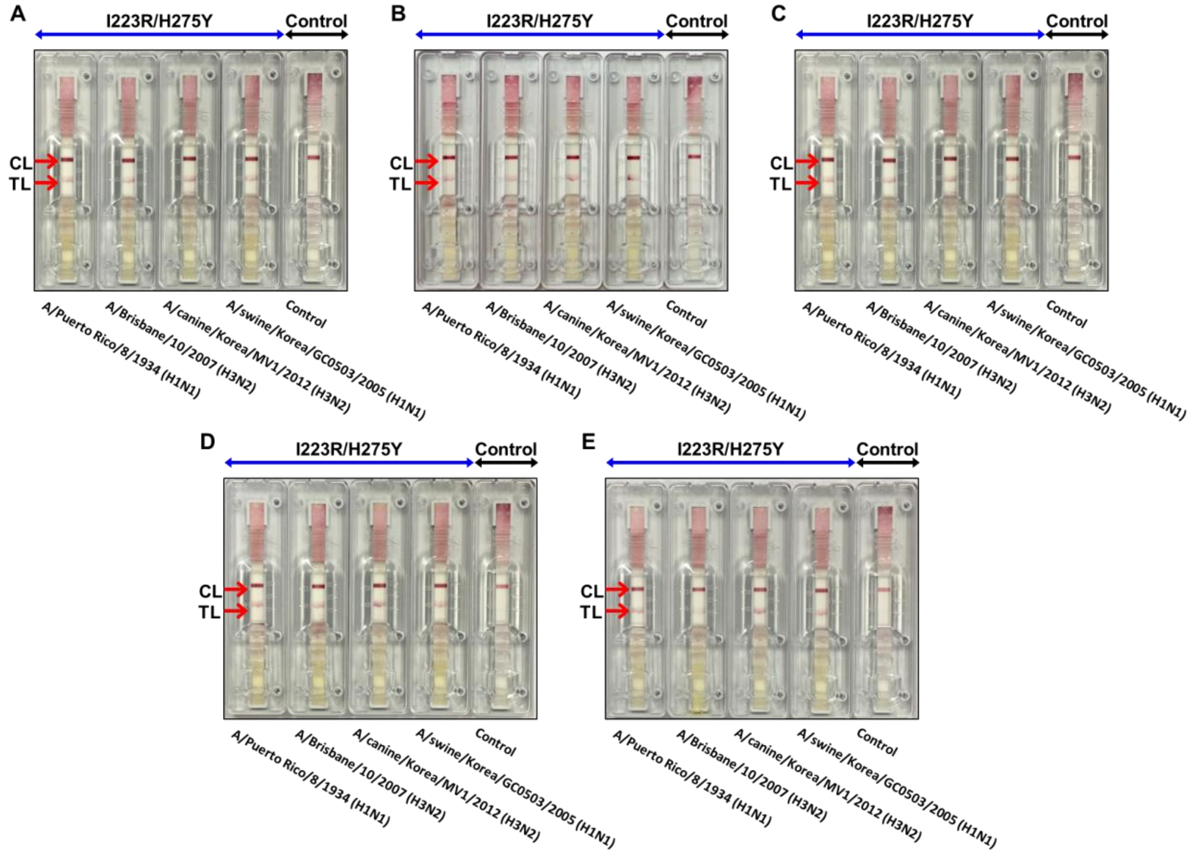

Supplementary Figure 13. (A-E) Optical images of A4-based lateral flow systems after detection of influenza-positive nasopharyngeal swab samples in the absence or presence of I223R/H275Y influenza viruses (A/Puerto Rico/8/1934 (H1N1), A/Brisbane/10/2007 (H3N2), A/swine/Korea/GC0503/2005 (H1N1), and A/canine/Korea/MV1/2012 (H3N2)).
